# Anticholinergic burden in the German National Cohort (NAKO): Patterns of anticholinergic drug exposure and utilization

**DOI:** 10.1016/j.rcsop.2026.100869

**Published:** 2026-10-23

**Authors:** Julie K. Könnecke, Nele L. Bößneck, Alexandra Drebka, Annika J. Scholl, Miriam Degen, Nina Ebert, Margit Heier, Thomas Keil, Michael Leitzmann, Rafael Mikolajczyk, Ilais Moreno Velásquez, Nadia Obi, Phil Pendt, Tobias Pischon, Nahlah Sallam, Ben Schöttker, Margarethe Woeckel, Beatrice E. Bachmeier

**Affiliations:** a Institute of Pharmaceutical Biology, Goethe University Frankfurt, Frankfurt am Main, Germany; b Division of Clinical Epidemiology of Early Cancer Detection, German Cancer Research Center, Heidelberg, Germany; c Institute for Biometrics and Epidemiology, German Diabetes Center, Leibniz Center for Diabetes Research at Heinrich Heine University Düsseldorf, Düsseldorf, Germany; d Institute of Epidemiology, Helmholtz Munich, German Research Center for Environmental Health (GmbH), Neuherberg, Germany; e KORA Study Center, University Hospital Augsburg, Augsburg, Germany; f Institute of Social Medicine, Epidemiology and Health Economics, Charité - University Medicine Berlin, Berlin, Germany; g Institute of Clinical Epidemiology and Biometry, University of Würzburg, Würzburg, Germany; h State Institute of Health I, Bavarian Health and Food Safety Authority, Erlangen, Germany; i Department of Epidemiology and Preventive Medicine, University of Regensburg, Regensburg, Germany; j Institute for Medical Epidemiology, Biometrics and Informatics, Interdisciplinary Center for Health Sciences, Medical Faculty, Martin Luther University Halle-Wittenberg, Halle (Saale), Germany; k Molecular Epidemiology Research Group, Max Delbrück Center for Molecular Medicine in the Helmholtz Association, Berlin, Germany; l Institute for Occupational and Maritime Medicine, University Medical Center Hamburg-Eppendorf, Hamburg, Germany; m Institute for Community Medicine, University Medicine Greifswald, Greifswald, Germany; n Biobank Technology Platform, Max Delbrück Center for Molecular Medicine in the Helmholtz Association, Berlin, Germany; o Charité - University Medicine Berlin, Corporate Member of Freie Universität Berlin and Humboldt-Universität zu Berlin, Berlin, Germany; p Institute for Medical Information Processing, Biometry, and Epidemiology, Faculty of Medicine, LMU Munich, Munich, Germany; q Department of Psychiatry and Psychotherapy, LMU University Hospital, Ludwig Maximilian University of Munich, Munich, Germany

**Keywords:** Anticholinergic burden, Pharmacoepidemiology, Polypharmacy, Over-the-counter medication, German National Cohort

## Abstract

**Background:**

Medication-related anticholinergic burden (AB) has been studied mainly in older adults and selected patient populations, whereas population-based evidence across adulthood remains limited, particularly regarding over-the-counter (OTC) drug use and utilization patterns.

**Objective:**

To characterize the frequency, distribution, and pharmacological drivers of AB in the German National Cohort (NAKO).

**Methods:**

We conducted a cross-sectional analysis of NAKO baseline data collected in 2014–2019 from participants aged 19–75 years recruited through age- and sex-stratified random sampling from population registries. AB was quantified from participant-reported seven-day medication use using the German Anticholinergic Burden Scale; cumulative scores ≥ 1 and ≥ 3 defined any and high burden, respectively. Descriptive analyses characterized AB by age, sex, drug class, prescription status, and utilization pattern; logistic regression assessed associations of AB with age, sex, and polypharmacy.

**Results:**

Among 199,813 participants (median age 51 years; 50.4% women), 17.4% had any and 3.0% had high AB, with exposure in all analyzed age groups. Any and high AB were associated with female sex (aOR = 1.09, 95% CI 1.06–1.12 and aOR = 1.44, 95% CI 1.37–1.53, respectively), most older age groups, and polypharmacy. Nervous system drugs contributed most to aggregate AB. Medications obtained OTC accounted for over one tenth of the sample's burden, with a greater relative contribution in younger age groups. Most anticholinergic drugs were used on a scheduled basis, with daily and long-term use predominating.

**Conclusion:**

In this large population-based cohort, anticholinergic exposure was observed across all examined age groups, and OTC medications made a non-negligible contribution to the overall burden. These findings suggest that age range and non-prescription medication use may be important considerations when assessing AB.

## Highlights

• Anticholinergic burden was identified in 17.4% of NAKO participants.
• Exposure occurred in all analyzed age groups and was more frequent at older ages.
• Women and participants with polypharmacy had higher odds of both any and high burden.
• Nervous system drugs accounted for the largest share of aggregate burden.
• Drugs obtained OTC, mainly systemic antihistamines, contributed notably to exposure.

## List of abbreviations

**Table:** 

AB	Anticholinergic burden
AD(s)	Anticholinergic drug(s)
AI	Artificial intelligence
AMVV	Ordinance on Prescription-Only Medicinal Products (Arzneimittelverschreibungsverordnung)
aOR(s)	Adjusted odds ratio(s)
ATC	Anatomical Therapeutic Chemical
CI(s)	Confidence interval(s)
cOR(s)	Crude odds ratio(s)
CRediT	Contributor Role Taxonomy
excl.	Excluding
Fig.	Figure
GABS	German Anticholinergic Burden Scale
IDOM	Instrument for Database-assisted Online recording for Medication
IQR	Interquartile range
NAKO	German National Cohort
OTC	Over-the-counter
PRN	Pro re nata
PZN(s)	Pharmaceutical Central Number(s)
Rx	Prescription
STROBE	Strengthening the reporting of observational studies in epidemiology

## 1 Introduction

Anticholinergic drugs (ADs) are used across a wide range of therapeutic areas, including chronic obstructive pulmonary disease, depression, Parkinson's disease, and overactive bladder.^1^, ^2^, ^3^ Their pharmacological action involves antagonism at muscarinic acetylcholine receptors, which can lead to both central and peripheral adverse effects.^3^ Anticholinergic burden (AB) refers to the cumulative anticholinergic exposure associated with an individual's medication regimen. Typically estimated from the number and potency of anticholinergic agents taken, AB serves as an indirect, medication-based indicator of potential anticholinergic effects.^4^, ^5^, ^6^ A high AB has frequently been associated with adverse effects and clinical outcomes, including cognitive impairment, falls, delirium, and increased mortality, particularly among older adults.^2^, ^7^, ^8^, ^9^, ^10^

Frequently reported contributors to AB include psychotropic medications, bladder antimuscarinics, antihistamines, and cardiovascular, respiratory and gastrointestinal drugs, with considerable variability across analyses and cohorts.^11^, ^12^, ^13^, ^14^, ^15^, ^16^, ^17^, ^18^, ^19^

Anticholinergic burden has been documented across diverse countries and healthcare settings, with implications for both clinical practice and public health.^11^, ^20^, ^21^, ^22^, ^23^ However, determining precise and comparable exposure rates is a major challenge, largely due to differences in methodology, national prescribing practices, and cohort profiles.^21^, ^24^, ^25^ Therefore, prevalence estimates vary substantially, ranging from approximately 10% to over 70% in some samples of older individuals.^10^, ^11^, ^12^, ^13^, ^26^, ^27^ Additionally, comparatively little is known about the distribution of AB in younger and middle-aged adults, as research on AB has been predominantly conducted in geriatric populations, which is clinically intuitive given the higher prevalence of multimorbidity and polypharmacy in this age group, increasing the likelihood of anticholinergic exposure.^14^, ^28^, ^29^, ^30^, ^31^ Furthermore, due to age-related changes in pharmacodynamics and pharmacokinetics, older adults are more susceptible to adverse drug effects.^32^, ^33^ While this heightened vulnerability justifies the focus on older adults, it does not preclude potentially clinically meaningful AB in younger and middle-aged individuals, particularly given emerging evidence linking earlier-life exposure to adverse outcomes, including in the neurocognitive domain.^34^, ^35^, ^36^, ^37^, ^38^ The few population-based analyses covering broader age ranges suggest that exposure to ADs extends across the life course, with prevalence estimates of about 20–40% reported in younger and middle-aged individuals, and approximately one third of the general population affected overall.^11^, ^13^ Notably, AB has repeatedly been found to be higher in women,^12^, ^13^ though this has rarely been examined beyond geriatric samples or secondary data sources.

Consistent with international literature, research from Germany has predominantly focused on geriatric and hospitalized populations or individuals with multimorbidity.^14^, ^39^, ^40^ A large-scale claims-based analysis extending across the full age range broadened this perspective at the population level; however, its reliance on administrative data precluded capturing detailed information on actual intake and real-world utilization patterns, as well as over-the-counter (OTC) medication use.^13^ The omission of OTC medications – particularly in claims-based data – reflects a broader limitation in anticholinergic exposure research, given their widespread use and the availability of several commonly used anticholinergic drugs (e.g., diphenhydramine and loratadine) without prescription.^41^, ^42^, ^43^, ^44^, ^45^

Several scoring systems have been developed to quantify AB by classifying drugs based on their anticholinergic potency.^8^ For medications authorized in Germany, Kiesel et al. (2018) developed the German Anticholinergic Burden Scale (GABS) based on a systematic literature review of existing scales and expert opinion.^42^ The GABS comprises a list of medications and assigns integer scores for anticholinergic activity ranging from 0 (no anticholinergic effects) to 3 (strong anticholinergic effects). Summing the respective scores of all ADs taken by an individual provides a measure for pharmacoepidemiological research and clinical practice.^8^, ^42^, ^46^, ^47^

To address the knowledge gaps in quantifying and characterizing AB across the adult age range, including OTC medication use and utilization patterns, we conducted a cross-sectional pharmacoepidemiological analysis using data from the German National Cohort (NAKO Gesundheitsstudie), a population-based study of approximately 200,000 individuals aged 19–75 years at baseline.^48^, ^49^, ^50^ Designed to investigate causes of major chronic diseases and associated risk factors,^49^ this cohort provides a unique framework for a comprehensive evaluation of anticholinergic exposure. Using the GABS, we aimed to examine the frequency and composition of AB by age and sex, its pharmacological drivers among prescription and non-prescription drugs, and utilization patterns of ADs, providing a reference for future research on lifestyle and healthcare correlates, AB trajectories, and potential associations with adverse health outcomes.

## 2 Methods

### 2.1 Data source and study population

We conducted a cross-sectional analysis of pseudonymized baseline data from the NAKO, a large population-based prospective study.^48^ The baseline data were collected between 2014 and 2019. Participants aged 19 to 75 years were recruited at 18 study centers across Germany from age- and sex-stratified random samples drawn from population registries.^48^ Registry information on sex could be updated at participants' request during the baseline assessment. Data collection took place during baseline study center visits and included physical and medical examinations, self-administered questionnaires, and computer-assisted interviews conducted by trained study personnel according to standardized study protocols.^48^ The detailed study design has been described elsewhere.^48^, ^49^, ^50^, ^51^ The NAKO was approved by all local ethics committees and is conducted in accordance with national legislation and the Declaration of Helsinki (1975, as revised). All participants gave written informed consent.^48^, ^49^

The present analysis was reported in accordance with the Strengthening the reporting of observational studies in epidemiology (STROBE) statement.^52^ An overview of the study methodology is presented in Fig. S1.

### 2.2 Medication assessment

Medication use during the seven days preceding each participant's baseline assessment was recorded in computer-assisted face-to-face interviews using the IDOM (Instrument for Database-assisted Online recording for Medication) software, specifically developed for the standardized and comprehensive documentation of all medications reported by participants.^48^, ^53^, ^54^ Participants were asked to bring medication packages to the study center visit to support identification and recording of the products (“brown bag review”), with telephone follow-up when packages had been forgotten. For both prescription and OTC preparations, Pharmaceutical Central Numbers (PZNs) were mapped to the May 2018 version of the German adaptation of the Anatomical Therapeutic Chemical (ATC) classification,^55^ and detailed information on drug utilization patterns was collected.^54^ OTC drugs were defined as all medications documented as non-prescribed, whether self-selected or purchased following a physician's recommendation; medications available OTC but documented as prescribed were not included in this category. For entries initially reported as obtained OTC but incompatible with German prescribing regulations at the time of baseline examination, i.e., drugs that legally required a prescription as per the Ordinance on Prescription-Only Medicinal Products (Arzneimittelverschreibungsverordnung, AMVV) § 1, manual reassignment to the prescription category was performed.

The medication count comprised all preparations documented and retained in the quality-assessed IDOM dataset, including dietary supplements where entered. For pragmatic reasons, this count of documented preparations or active ingredients is referred to as the number of medications. Using a commonly applied threshold, polypharmacy was defined as the use of five or more medications.^56^, ^57^

### 2.3 Anticholinergic burden

Anticholinergic burden was calculated according to the German Anticholinergic Burden Scale (GABS).^42^ This instrument, developed via systematic literature research on previous scales and expert opinion, scores 504 medications authorized in Germany from 0 (no anticholinergic effects) to 3 (strong anticholinergic effects) based on pharmacological properties and clinical evidence.^5^, ^42^, ^58^

For each participant, the GABS scores of the reported medications were summed to obtain the individual total anticholinergic burden score as an indicator of cumulative medication-related anticholinergic potential. All participant-reported medications without a corresponding GABS rating were assigned a score of 0. Cumulative burden scores were categorized as 0, 1, 2, or ≥ 3. A cumulative score of ≥ 3 was classified as a high anticholinergic burden, consistent with previous research.^13^, ^39^, ^42^, ^59^, ^60^ To avoid multiple counting of the same substance, scores were derived by including each distinct full-length (5th level) ATC code only once per individual, irrespective of multiple brands, formulations, or dosages; this approach was applied consistently to both burden-score calculation and medication-level analyses.

### 2.4 Statistical analysis

Descriptive statistics were applied to characterize participant demographics and to estimate the frequency and extent of anticholinergic exposure overall and across subgroups. Crude and adjusted logistic regression analyses were conducted as supportive analyses. Two separate binary outcomes – any anticholinergic burden, defined as a GABS score ≥ 1 versus 0, and high anticholinergic burden, defined as a GABS score ≥ 3 versus < 3 – were analyzed to describe their cross-sectional associations with age group, sex, and polypharmacy status. Adjusted models additionally included research site. Results are reported as crude and adjusted odds ratios (ORs) with 95% confidence intervals (CIs). Interpretation focused on the magnitude and uncertainty of the estimated associations, without dichotomizing results by their statistical significance.^61^ Due to the structural relationship between AB and overall medication use, sensitivity analyses excluding polypharmacy from the regression models were conducted to assess its influence on the estimated associations of sex and age with AB. Models were also fitted with the number of non-anticholinergic medications, defined as those not contributing to AB, in place of polypharmacy to account for concurrent medication use while reducing the direct overlap with the outcomes. Apparent model discrimination was assessed using the c-statistic (area under the receiver operating characteristic curve) with 95% CIs.

The relative frequency of individual anticholinergic drugs or drug classes was calculated by dividing the number of corresponding ADs by the total number of ADs within the respective (sub-)sample. To assess the influence of considering OTC medication use on AB ascertainment, the distributions of no AD use, Rx-only AD use, combined Rx and OTC AD use, OTC-only AD use, and AD use with unknown prescription status were determined in the total sample and among participants with AB > 0. Aggregate AB within the overall sample or a specified stratum was calculated by summing the participant-level cumulative GABS scores; for comparisons between strata, aggregate AB was additionally standardized per 1,000 participants. The relative contribution of ATC drug classes to the sample's aggregate AB was calculated as the proportion of burden attributable to each ATC class.

All analyses were performed using R (v4.5.1) with RStudio (v2025.09.2) or IBM SPSS Statistics (v31.0.0.0). Generative artificial intelligence (AI) tools (OpenAI's ChatGPT, v5.2–5.5, and Anthropic's Claude, Sonnet 4.6 and 5) were used to assist with code development and debugging. Tables and figures were generated using Microsoft Office 365 (including the think-cell 14 add-in) and GraphPad Prism (v10.5.0).

## 3 Results

### 3.1 Selection and characterization of the study sample

Of the 204,611 NAKO participants with active consent at the time of data transfer (March 2026), those with missing data on age, sex, or medication use, and those with potentially erroneous medication entries were excluded (*n* = 4,798, Fig. 1).

**Fig. 1:**
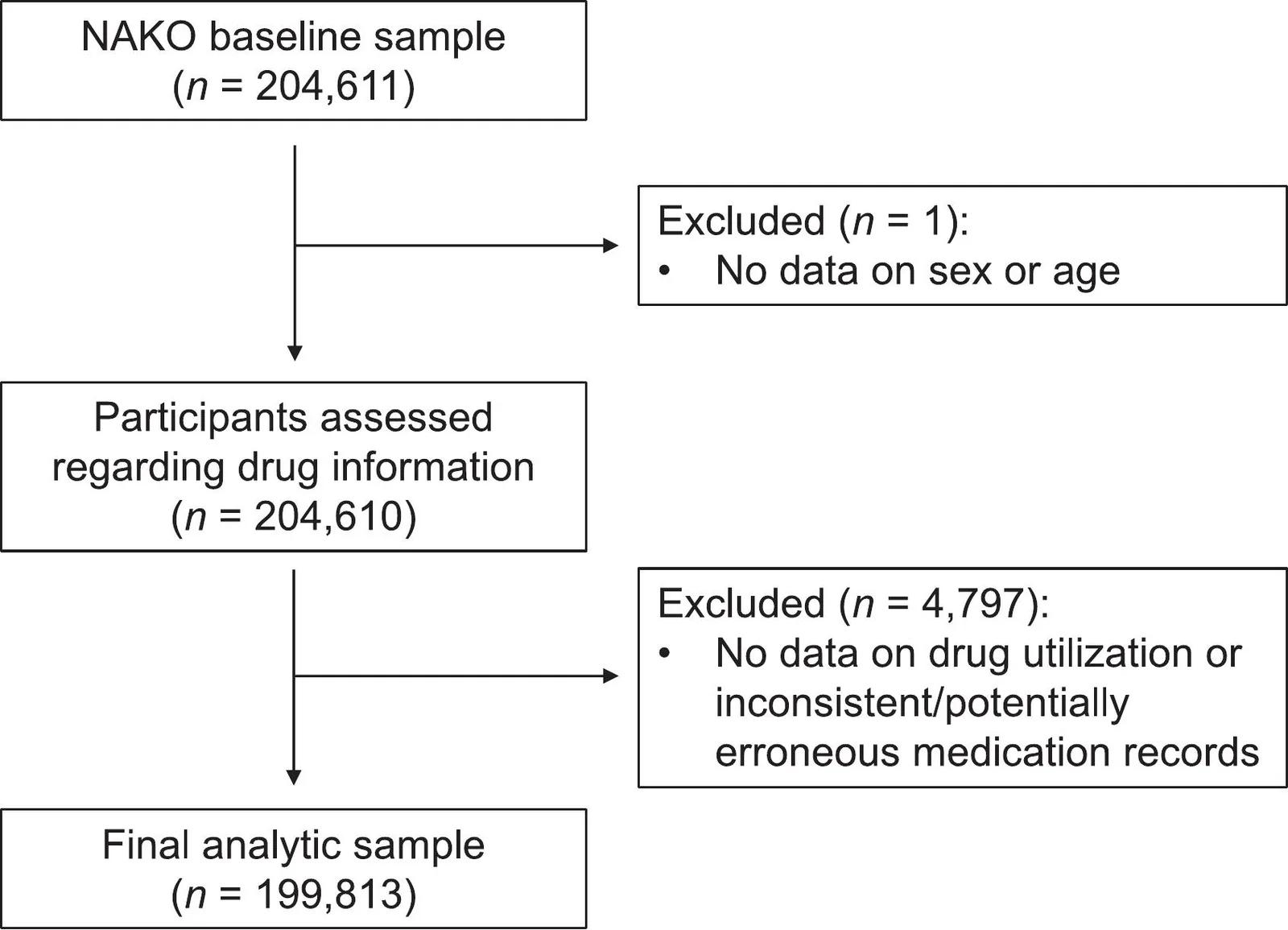
STROBE flowchart showing the exclusion process for constructing the final analytic sample. NAKO baseline examination participants were excluded due to missing demographic information (sex or age), missing information on medication exposure or count, or if their medication documentation was judged ambiguous or potentially erroneous based on NAKO quality assessment procedures.

The median age of the analytic sample was 51 years (IQR 42–60), and the overall sex distribution was balanced, with 50.4% women and 49.6% men. The median number of self-reported overall medications was 2 (IQR 0–3) (Table 1), and 16.2% of participants used ≥ 5 medications.

**Table 1:** Profile of the analytic sample regarding demographic and drug exposure variables (overall and by AB category).

	Total	GABS score = 0	GABS score = 1	GABS score = 2	GABS score ≥ 3
Number of individuals	199,813 (100.0)	165,059 (82.6)	23,258 (11.6)	5,578 (2.8)	5,918 (3.0)
Age [years]	51 (42–60)	50 (41–59)	56 (47–64)	57 (49–64)	57 (49–64)
Age group					
≤ 29 years	19,585 (9.8)	17,948 (10.9)	1,223 (5.3)	206 (3.7)	208 (3.5)
30–39 years	21,173 (10.6)	19,159 (11.6)	1,480 (6.4)	272 (4.9)	262 (4.4)
40–49 years	51,845 (25.9)	45,030 (27.3)	4,738 (20.4)	1,048 (18.8)	1,029 (17.4)
50–59 years	53,468 (26.8)	43,404 (26.3)	6,509 (28.0)	1,640 (29.4)	1,915 (32.4)
60–69 years	49,474 (24.8)	36,517 (22.1)	8,470 (36.4)	2,186 (39.2)	2,301 (38.9)
≥ 70 years	4,268 (2.1)	3,001 (1.8)	838 (3.6)	226 (4.1)	203 (3.4)
Sex					
Female	100,803 (50.4)	81,753 (49.5)	12,255 (52.7)	3,047 (54.6)	3,748 (63.3)
Male	99,010 (49.6)	83,306 (50.5)	11,003 (47.3)	2,531 (45.4)	2,170 (36.7)
Number of medications	2 (0–3)	1 (0–3)	4 (2–6)	5 (3–8)	6 (4–9)
Polypharmacy					
No	167,415 (83.8)	149,093 (90.3)	14,134 (60.8)	2,274 (40.8)	1,914 (32.3)
Yes	32,398 (16.2)	15,966 (9.7)	9,124 (39.2)	3,304 (59.2)	4,004 (67.7)

Values are expressed as median (IQR) for continuous variables and n (%) for categorical variables. AB, anticholinergic burden; GABS, German Anticholinergic Burden Scale; IQR, interquartile range.

Within the seven days preceding the baseline examination, 17.4% of individuals in the sample reported the use of at least one drug with anticholinergic potency listed in the GABS (score ≥ 1). Among those exposed individuals, the majority (66.9%) exhibited a low cumulative score of 1, while moderate and high AB were roughly equally distributed (16.0% and 17.0%; score = 2 or ≥ 3, respectively). In the overall study population, 11.6% had a low, 2.8% a moderate, and 3.0% a high AB (Table 1, Fig. 2).

**Fig. 2:**
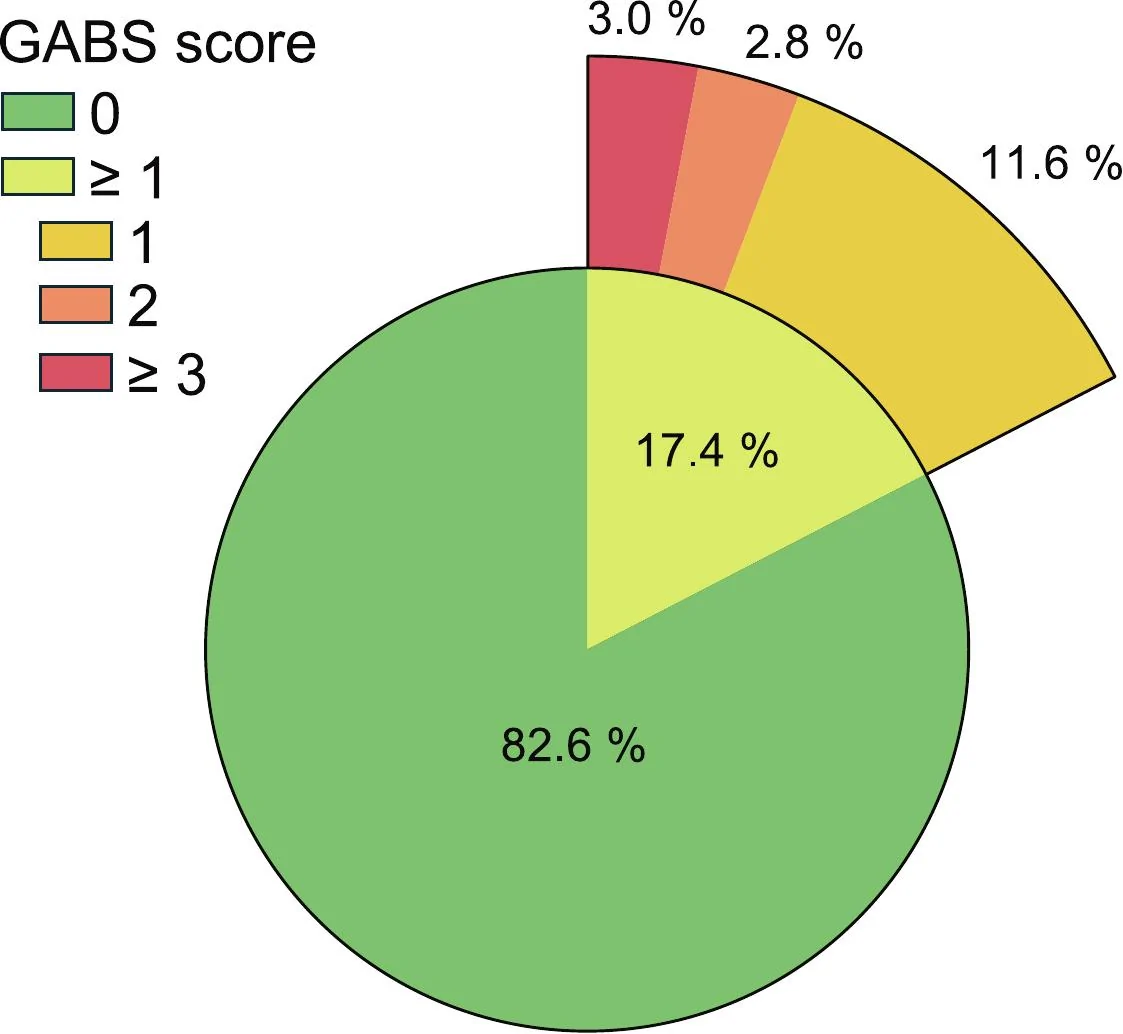
Relative frequency and magnitude of AB in the study population. AB, anticholinergic burden; GABS, German Anticholinergic Burden Scale.

Higher burden levels were accompanied by gradual differences in participant characteristics (Table 1). Median age increased across GABS score categories, from 50 years (IQR 41–59) among individuals with a score of 0 to 57 years (IQR 49–64) among those with scores of 2 or ≥ 3. Similarly, the proportion of women was elevated in higher burden categories, reaching 63.3% in the high-burden group. Overall medication use also increased steadily across burden categories, from a median of 1 medication (IQR 0–3) among individuals with no burden to 6 medications (IQR 4–9) among those with high AB, with the proportion of participants with polypharmacy rising from 9.7% to more than two thirds.

### 3.2 Patterns of anticholinergic burden

Anticholinergic exposure was slightly more common among women than men overall (18.9% versus 15.9%), and both sexes showed a rising trend in frequency of exposure to ADs with increasing age. Fewer than one in ten participants younger than 30 years had any AB, compared with approximately one third of those aged 70 years or older, corresponding to a more than threefold higher crude proportion in the oldest age group. Across age groups, women consistently showed higher frequencies of exposure to anticholinergic medication than men (Fig. 3A). Similarly, in those exposed to ADs, the magnitude of burden was higher in women (mean overall GABS scores of 1.69 ± 1.16) compared with men (1.53 ± 1.03). Within the subgroup with AB, mean burden scores showed modest variation by age group, peaking in the 50–59-year age group (1.69 ± 1.18), with a maximum difference of approximately 0.26 points between the highest and lowest age group averages (Fig. 3B).

**Fig. 3:**
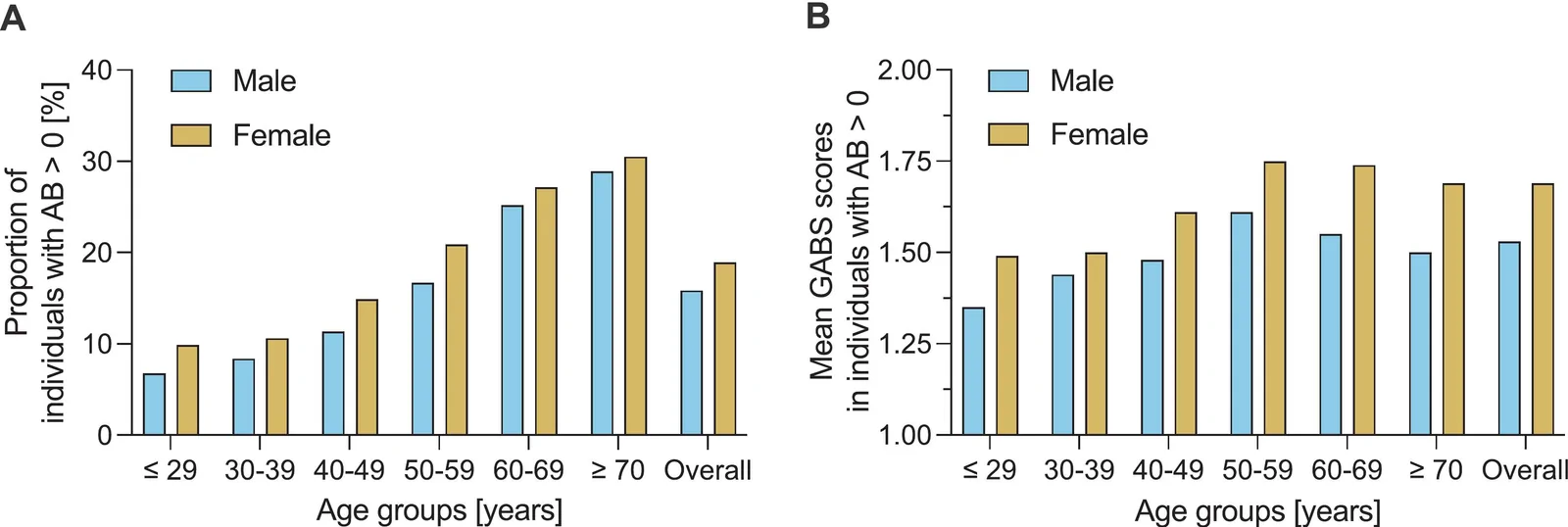
AB by age group and sex. (A) Proportion of participants exposed to at least one AD (i.e., AB > 0) stratified by age group and sex. (B) Mean GABS scores among those exposed to ADs stratified by age group and sex. AB, anticholinergic burden; AD, anticholinergic drug; GABS, German Anticholinergic Burden Scale.

Consistent with the descriptive patterns, crude and adjusted logistic regression analyses indicated that the odds of both any and high AB (GABS scores ≥ 1 and ≥ 3, respectively) varied by sex, age group, and extent of overall medication use (Table 2). Compared to men, women had higher adjusted odds of both any AB (aOR = 1.09, 95% CI 1.06–1.12) and high AB (aOR = 1.44, 95% CI 1.37–1.53). The odds of any AB increased across age groups and remained elevated after adjustment, peaking in the 60–69-year age group (aOR = 2.19, 95% CI 2.06–2.31) compared to those aged ≤ 29 years. For high AB, adjusted odds were elevated from age 40–49 years onward, with the highest odds observed among participants aged 50–59 years (aOR = 1.98, 95% CI 1.71–2.29). Polypharmacy showed the strongest association with anticholinergic exposure. After adjustment, participants using ≥ 5 medications had substantially higher odds of any AB (aOR = 7.22, 95% CI 7.03–7.43) and high AB (aOR = 10.75, 95% CI 10.13–11.40) than those without polypharmacy.

**Table 2:** Crude and adjusted odds ratios for any and high anticholinergic burden by sex, age group, and polypharmacy status.

	Any burden (GABS score ≥ 1)		High burden (GABS score ≥ 3)	
	cOR (95% CI)	aOR (95% CI)	cOR (95% CI)	aOR (95% CI)
**Sex**				
**Male**	Ref.	Ref.	Ref.	Ref.
**Female**	1.24 (1.21–1.27)	1.09 (1.06–1.12)	1.72 (1.63–1.82)	1.44 (1.37–1.53)
**Age group**				
**≤ 29 years**	Ref.	Ref.	Ref.	Ref.
**30–39 years**	1.15 (1.08–1.23)	1.08 (1.00–1.15)	1.17 (0.97–1.40)	1.03 (0.85–1.24)
**40–49 years**	1.66 (1.57–1.76)	1.41 (1.33–1.49)	1.89 (1.62–2.19)	1.38 (1.19–1.61)
**50–59 years**	2.54 (2.41–2.69)	1.86 (1.75–1.97)	3.46 (3.00–4.00)	1.98 (1.71–2.29)
**60–69 years**	3.89 (3.68–4.11)	2.19 (2.06–2.31)	4.54 (3.94–5.24)	1.78 (1.53–2.06)
**≥ 70 years**	4.63 (4.26–5.03)	2.13 (1.95–2.34)	4.65 (3.82–5.66)	1.46 (1.19–1.78)
**Polypharmacy**				
**No**	Ref.	Ref.	Ref.	Ref.
**Yes**	8.37 (8.15–8.60)	7.22 (7.03–7.43)	12.19 (11.53–12.89)	10.75 (10.13–11.40)

cOR, crude odds ratio; aOR, adjusted odds ratio (adjusted for sex, age group, polypharmacy, and research site); CI, confidence interval; GABS, German Anticholinergic Burden Scale; Ref., reference.

Sensitivity analyses excluding polypharmacy yielded directionally consistent but larger aORs for sex and age, with age-group estimates increasing monotonically (Table S1). In the sensitivity models replacing polypharmacy with the non-anticholinergic medication count, each additional non-anticholinergic medication was associated with higher odds of any AB (aOR = 1.30, 95% CI 1.30–1.31) and high AB (aOR = 1.28, 95% CI 1.27–1.29; Table S2). Sex estimates were similar to those in the main models, whereas age-group aORs were higher for both outcomes. C-statistics for the main and sensitivity models are presented in Table S3.

### 3.3 Most commonly used ADs and major contributing drug classes by ATC category

Within the analytic sample, 136 of the 151 medications classified as anticholinergic (GABS score ≥ 1) by Kiesel et al.^42^ were represented.

Metoprolol, metformin, and cetirizine were the most frequently used ADs in the cohort, accounting for 17.8%, 11.4%, and 7.6% of ADs reported, respectively (Fig. 4A). The most commonly used ADs stratified by assigned GABS score were metoprolol (score of 1), opipramol (score of 2), and amitriptyline (score of 3), as specified in Table S4. Among individuals with high AB, the use of the high-potency AD amitriptyline was most frequently reported (Fig. S2).

**Fig. 4:**
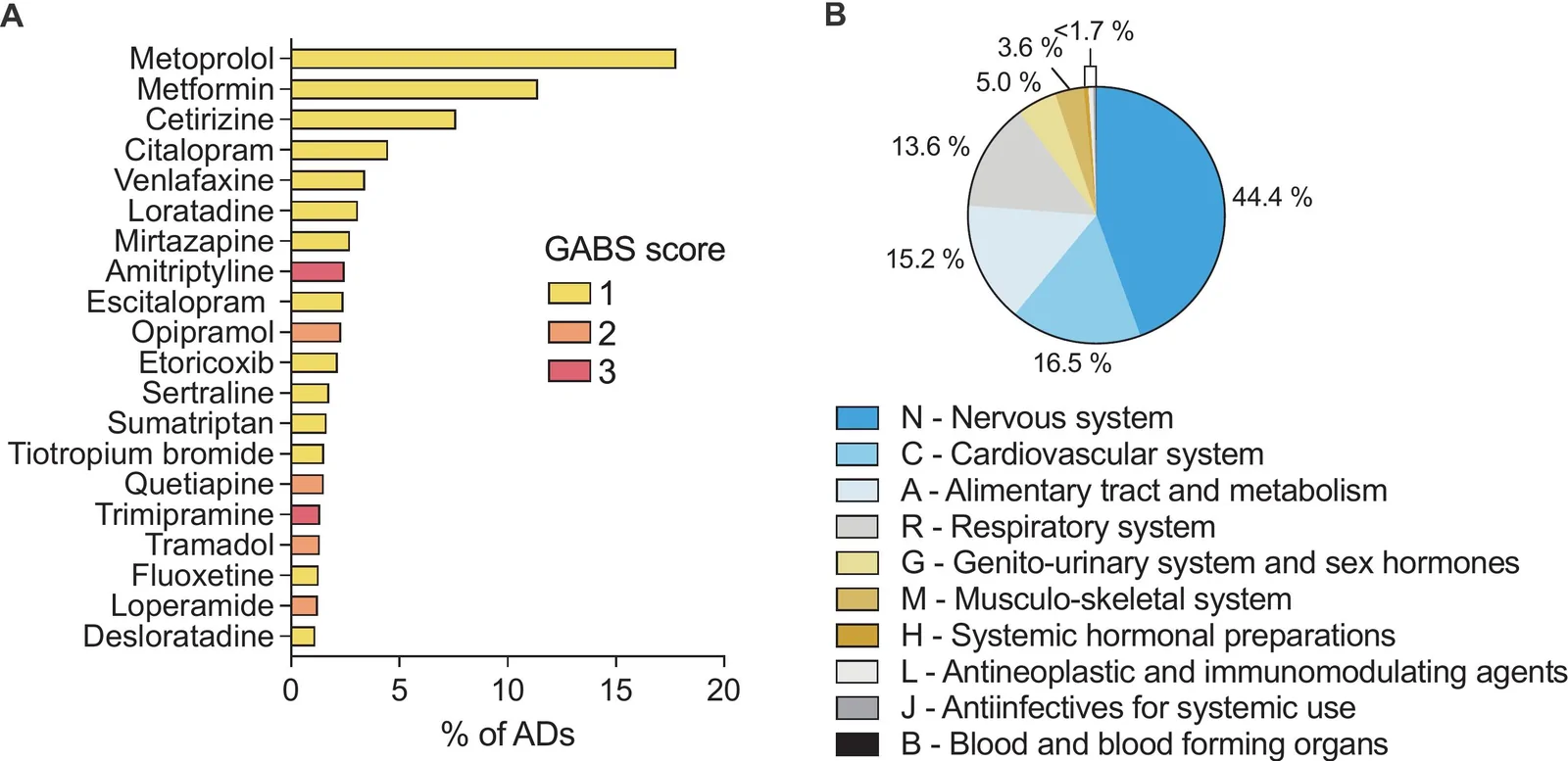
Drugs and drug classes contributing to the sample's overall AB. (A) Top 20 most frequently used ADs in the cohort, displayed as their share of all ADs recorded. Bar colors indicate anticholinergic potency as per the GABS (score 1 (yellow), 2 (orange), or 3 (red)). (B) Contribution of different ATC categories to the aggregate AB, depicted as the share of the total summed GABS scores attributable to the respective 1st level ATC classifications. ATC, Anatomical Therapeutic Chemical; AB, anticholinergic burden; ADs, anticholinergic drugs; GABS, German Anticholinergic Burden Scale. (For interpretation of the references to color in this figure legend, the reader is referred to the web version of this article.)

Across the main ATC categories, the largest contributions to the sample's aggregate AB came from drugs classified under the nervous system (N; 44.4%), cardiovascular system (C; 16.5%), alimentary tract and metabolism (A; 15.2%), and respiratory system (R; 13.6%) categories (Fig. 4B).

### 3.4 Role of OTC medications

Compared with ascertainment based on AD use regardless of prescription status, exclusion of OTC ADs reduced the proportion of participants classified with any AB (AB > 0) by approximately 2.1 percentage points. Among participants with any AB, 14.7% had an OTC component to their burden, including 12.2% with OTC AD-only use and 2.5% with combined prescription and OTC AD use. Under prescription-only ascertainment, these groups corresponded to complete non-ascertainment of AB and partial underestimation of AB, respectively (Fig. S3).

The OTC-attributable share of aggregate AB decreased with age in both sexes, from approximately 40% among participants aged ≤ 29 years to less than 5% among those aged ≥ 70 years. Overall, OTC ADs accounted for about a tenth of aggregate AB in both men and women (Fig. 5).

**Fig. 5:**
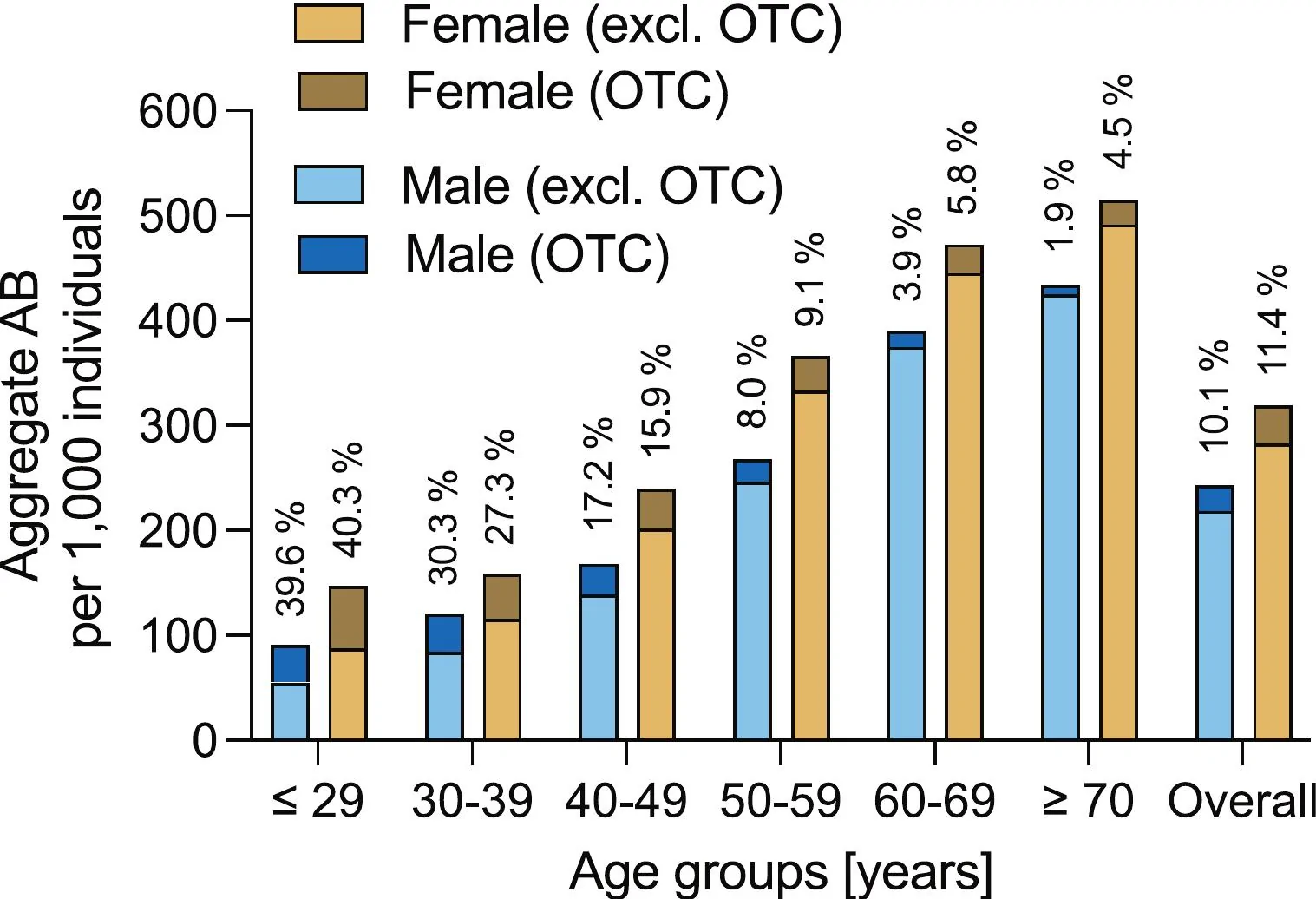
OTC contribution to AB by age group and sex. Stacked bars represent aggregate GABS score points per 1,000 participants within each stratum; the overall category includes all age groups. AB excluding OTC comprises burden attributable to prescription and unclassified ADs. Percentages indicate the share of aggregate AB attributable to OTC ADs. AB, anticholinergic burden; ADs, anticholinergic drugs; excl., excluding; GABS, German Anticholinergic Burden Scale; OTC, over-the-counter.

Across all reported ADs, 11.7% were classified as OTC. The proportion classified as OTC was highest among low-potency ADs (GABS score 1) and declined with increasing anticholinergic potency (Fig. S4).

After grouping all ADs identified as OTC drugs at the 3rd level of the ATC classification, we found that antihistamines for systemic use (R06A) were by far the most frequently reported group in the analyzed sample (78.7% of OTC ADs). With about 7.2%, the next most common pharmacological subgroup was antipropulsives (A07D), followed by antiemetics and antinauseants (A04A), drugs for constipation (A06A), and antimigraine preparations (N02C), each contributing only 3–5%. All remaining anticholinergic OTC drugs combined accounted for less than 2.7% (Table 3).

**Table 3:** Most frequent 3rd level ATC categories among anticholinergic OTC medications used in the study population.

ATC category	% of OTC ADs	Main agent
R06A (antihistamines for systemic use)	78.7	Cetirizine
A07D (antipropulsives)	7.2	Loperamide
A04A (antiemetics and antinauseants)	4.6	Dimenhydrinate
A06A (drugs for constipation)	3.7	Bisacodyl
N02C (antimigraine preparations)	3.2	Naratriptan
Others	< 2.7	Dextromethorphan

Main agent denotes the most commonly reported OTC AD per category. ADs, anticholinergic drugs; ATC, Anatomical Therapeutic Chemical; OTC, over-the-counter.

### 3.5 Utilization patterns of ADs

Most anticholinergic agents were reported as being taken on a scheduled basis, rather than as needed (Fig. 6A), with pro re nata (PRN) use most often reported for systemic antihistamines and antimigraine preparations. ADs utilized on a scheduled regimen were taken predominantly daily (97.8%, Fig. 6B), and in most cases for 1 year or longer (Fig. 6C).

**Fig. 6:**
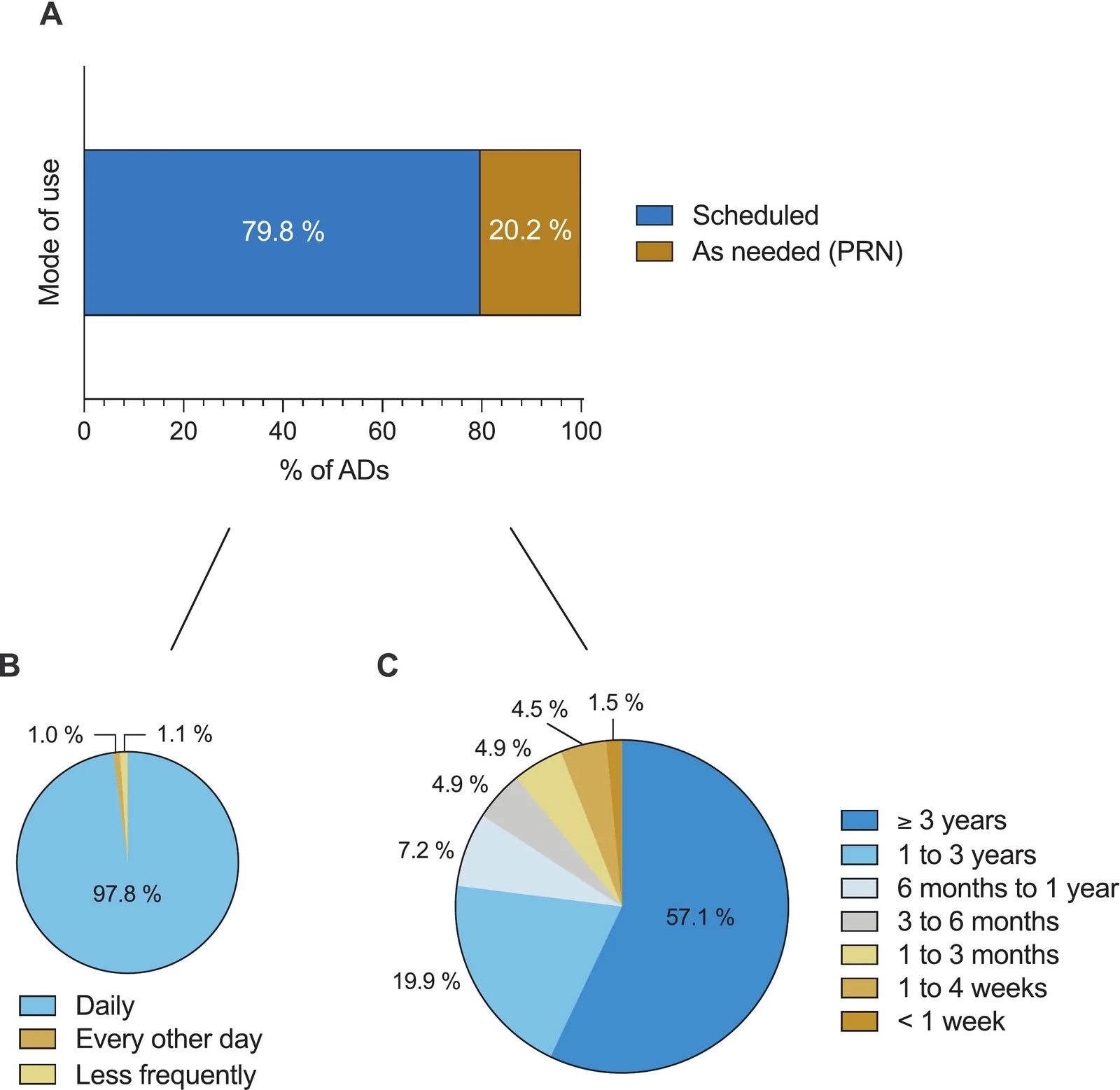
Utilization patterns of ADs. (A) Proportions of ADs used on a scheduled vs. as-needed (PRN) basis are shown. (B) Intake frequency and (C) duration of use reported for ADs used on a scheduled regimen. Information for each utilization variable was unavailable for < 0.6% of ADs; these records were excluded from the respective analyses. ADs, anticholinergic drugs; PRN, pro re nata.

## 4 Discussion

This large population-based analysis identified anticholinergic burden (AB) in a relevant proportion of participants and across all examined age groups. The odds of any and high AB were increased among women, older participants, and individuals with polypharmacy. Aggregate AB was mainly driven by drugs targeting the nervous, cardiovascular, alimentary/metabolic, and respiratory systems, while OTC medications – predominantly systemic antihistamines – contributed to the observed exposure pattern. Most ADs were reported to be used on a scheduled rather than as-needed basis, with daily and long-term use predominating among scheduled regimens.

By analyzing self-reported data on prescribed and non-prescribed medication use from participants in the NAKO, this analysis captured recent anticholinergic exposure across a broad adult age range. AB was quantified using the German Anticholinergic Burden Scale (GABS), a composite tool tailored to medications available in the German healthcare system and previously rated favorably in methodological evaluations.^5^, ^42^, ^58^, ^62^ These sample- and method-related features provide important context for interpreting our results in relation to other studies, as estimates of AB may vary substantially according to sample profiles, country-specific prescribing practices, medication availability, and healthcare system characteristics. In addition, methodological heterogeneity – including the use of different quantification tools, varying exposure windows, and differences in underlying data sources – can further affect cross-study comparisons.

Against this background, AB prevalence estimates in several international population-based studies restricted to older adults were higher than the overall NAKO estimate, although differences in age ranges and stratification may limit direct comparability. Available age-stratified estimates tended to be closer to those for older NAKO age groups.^63^, ^64^, ^65^, ^66^ The overall difference may therefore partly reflect the younger age distribution of the present cohort but may also be influenced by the contextual and methodological factors outlined above.

Comparisons with studies including younger or middle-aged adults were more informative but still showed some variation. A Finnish population-based study of middle-aged adults reported estimates comparable to ours, with 15% classified as having any AB and 3% as having high AB.^67^ In contrast, estimates were higher in Slovenian claims data among adults aged 19–64 years, and in German claims data across the full age range, where approximately one quarter to one third of individuals were classified as exposed.^11^, ^13^ The higher proportions of exposed individuals in the administrative data-based analyses, despite the absence of OTC medications, likely reflect the longer one-year observation period compared to the brief recall window applied in the NAKO and may further be influenced by differences between routinely collected prescription data and self-reported medication use.^11^, ^13^, ^68^ Comparability is further limited by differences in the tools applied for AB quantification, particularly for the Finnish and Slovenian studies, whereas the German claims data analysis used the same instrument as the present study.^11^, ^13^, ^67^ Finally, differences in study populations may have contributed to within-country variation: While the NAKO excludes individuals at both ends of the age spectrum and may be affected by selection bias,^48^, ^50^ the German claims data analysis was restricted to statutorily insured individuals.^13^ Although privately insured persons represent only a comparatively small population segment, their somewhat healthier profile may have contributed to differences between estimates.^69^, ^70^, ^71^, ^72^

Despite differences in magnitude, our findings align with these data in suggesting that anticholinergic exposure is typically lower in younger samples than in geriatric samples, yet non-negligible. However, the clinical interpretation of anticholinergic burden in younger individuals remains largely uncertain, given that most scales have been developed and evaluated primarily in geriatric settings.^21^ In light of emerging evidence – albeit heterogeneous – suggesting an association of anticholinergic drug use in early and mid-adulthood, as well as of prolonged exposure, with impaired cognitive functioning or neurostructural changes,^35^, ^37^, ^73^, ^74^, ^75^, ^76^ these findings nevertheless support further attention to anticholinergic exposure before older age. High anticholinergic burden has also been linked to reduced health-related quality of life in comparatively young populations,^23^, ^77^ and typical side effects of anticholinergic medications and their potential downstream consequences, such as xerostomia and increased risk of caries, are not restricted to older adults.^67^, ^78^, ^79^, ^80^ Together, these observations suggest that anticholinergic exposure earlier in adulthood should be considered in future longitudinal research, while the interpretation of burden scores and thresholds in younger populations requires further clarification.

Consistent with previous national and international data,^11^, ^12^, ^13^, ^14^, ^81^, ^82^ we found higher AB in women than in men. This pattern may partly reflect the higher prevalence of some conditions commonly treated with (high-potency) anticholinergic agents in women – such as urinary incontinence or depression^83^, ^84^, ^85^ – as well as sex-specific prescribing practices and healthcare utilization.^86^, ^87^, ^88^ However, differences in outcomes related to anticholinergic exposure in women compared to men remain comparatively underexamined, especially considering established differences in drug metabolism between sexes.^32^, ^89^

The descriptive age- and sex-related patterns were further underlined by the complementary regression-based analyses. Women had higher adjusted odds of both any AB and high AB than men, although the magnitude of the association was rather small, particularly for any AB. Similarly, most older age groups were associated with both outcomes after accounting for sex, polypharmacy, and research site. Polypharmacy showed the strongest association with anticholinergic exposure, consistent with previous research reporting a positive relationship between polypharmacy and greater anticholinergic burden.^12^, ^90^, ^91^ However, this finding should be interpreted in light of their shared basis in medication use, as a higher medication count may increase the opportunity for anticholinergic exposure. When polypharmacy was omitted from the model, sex and age associations remained directionally consistent but increased in magnitude, with a more continuous gradient across age groups, suggesting that differences in overall medication use may contribute to the observed patterns. Higher non-anticholinergic medication counts were also associated with both any and high AB, which may partly reflect underlying morbidity and prescribing indications.

As expected from previous studies,^11^, ^13^, ^27^, ^64^, ^65^, ^82^, ^92^ drugs acting on the nervous, cardiovascular, and respiratory systems, as well as alimentary tract and metabolism medications, contributed most to aggregate AB in the NAKO sample. The prominence of nervous system drugs likely reflects the widespread use of psychotropic medications, several of which possess high anticholinergic potency (e.g., amitriptyline and trimipramine)^42^ and thus markedly elevate AB. Despite low individual GABS scores, cardiovascular medications such as metoprolol contributed considerably to overall AB due to the frequency of their use, reflecting the high prevalence of associated conditions.^93^, ^94^ Taken together, these observations indicate that much of the observed burden stems from drugs and drug classes whose anticholinergic effects are secondary pharmacological properties rather than the principal mechanism of therapeutic action.

As demonstrated by our findings, OTC medications constituted a notable share of both AD counts and aggregate AB in the sample, with their relative contribution to AB particularly pronounced among younger individuals. Albeit less frequently represented among highly anticholinergic drugs, OTC medications occurred across all levels of anticholinergic potency. While their omission produced only a small absolute change in the sample-level prevalence of any AB, a relevant proportion of participants classified as having AB in the comprehensive medication assessment would nonetheless have been misclassified under prescription-only data. Although often overlooked in pharmacoepidemiological studies, their consideration as a source of anticholinergic risk may provide a more comprehensive view of real-world exposure levels. This is particularly relevant given that self-medication is widespread in many countries,^95^, ^96^ with studies from Europe and North America indicating prevalence estimates of OTC drug use comparable to those of prescription drug use.^41^, ^43^ Moreover, OTC medication use is commonly undertaken without supervision by healthcare professionals and may involve inappropriate or misuse-related consumption patterns.^97^, ^98^ Consequently, OTC-related anticholinergic burden likely remains largely unrecognized in routine clinical care, underlining the importance of making this source of exposure visible and explicitly characterizing it in population-based research. However, the impact of including or omitting OTC medication use on estimates of anticholinergic exposure likely varies between countries and data sources, particularly with regard to national regulations governing OTC availability and, in claims data analyses, reimbursement structures. It may further be influenced by the age structure of the study population and the types of medication use included in the AB quantification, including whether both scheduled and as-needed use are considered.

As most of the anticholinergic OTC drugs used by NAKO participants were antihistamines, future analyses could explore potential seasonal variation in AB and its implications, for instance in relation to allergy seasons.

Importantly, the present analysis describes recent, GABS-defined anticholinergic medication use rather than inappropriate prescribing or clinical harm. Nevertheless, given existing evidence of negative outcomes associated with chronic AD exposure,^37^, ^76^, ^99^, ^100^, ^101^ the frequent reporting of scheduled and longer-term use of anticholinergic agents in this cohort may indicate exposure patterns that warrant further evaluation in longitudinal and clinically enriched analyses.

Strengths of this analysis include the large sample size and the multicenter design of the NAKO. Furthermore, the availability of participant-reported medication data, including OTC drug use, provided a more complete picture of real-world anticholinergic exposure. By applying the GABS, a nationally adapted instrument with comparatively broad drug coverage, anticholinergic medication use in the NAKO sample was likely captured more sensitively than with many other instruments. Nevertheless, several limitations should be considered. Due to selection bias and age restrictions, the NAKO sample does not entirely reflect the sociodemographic structure of the general German population.^50^ The reported unweighted prevalence estimates therefore pertain to the analytic sample and are best interpreted as descriptive frequencies within the cohort. Medication information obtained from participants may be affected by recall bias, potentially leading to misclassification or underreporting of medication use, although this was likely mitigated by the brown bag review. Deduplication of ATC codes may have influenced utilization estimates. For the regression-based analyses, the limited covariate adjustment increases the potential for residual confounding. Comorbidities and other health-related or sociodemographic factors not included in the models may affect the reported associations. Furthermore, the GABS was not specifically developed for population-based exposure assessment, and its omission of combination products and restriction to drugs available at the time of development may lead to an underestimation of anticholinergic burden. Moreover, anticholinergic burden was assessed using a scoring instrument rather than through the clinical assessment of anticholinergic effects; therefore, the presence or severity of potential clinical manifestations could not be determined. Broader limitations of scale-based AB assessment, including limited consideration of individual drug dosages and responses, have been discussed elsewhere.^102^ Lastly, anticholinergic burden was estimated using a single scale, and different instruments may yield divergent estimates.^103^

## 5 Conclusion

Anticholinergic burden is most prevalent and currently most frequently studied in older populations; however, our findings demonstrate the presence of AD exposure also in younger and middle-aged adults in a large population-based sample. Consistent with previous research, AB was more common among women and older age groups. With a notable share of anticholinergic agents being OTC medications, an important and often overlooked source of AB was highlighted. Furthermore, exposure in the cohort was largely driven by routinely used, long-term treatments across several major therapeutic classes rather than a narrow subset of specialized medications, reflecting the broad relevance of anticholinergic agents in everyday pharmacotherapy.

Together, these findings highlight a broader age coverage and the inclusion of OTC drugs as important considerations for more comprehensively capturing AB, both in clinical medication reviews and in research into its determinants and potential long-term implications across the life course.

Future longitudinal analyses within the NAKO, including younger and middle-aged adults, may allow for an assessment of temporal trends and potential associations between AB and adverse outcomes, such as cognitive and functional decline.

## Declaration of the use of generative AI and AI-assisted technologies

OpenAI's ChatGPT (v5.2–5.5) and Anthropic's Claude (Sonnet 4.6 and 5) were used to assist with coding and language optimization. The authors thoroughly reviewed, revised, and approved all artificial intelligence (AI)-supported elements and remain fully responsible for the final content.

## CRediT authorship contribution statement

**Julie K. Könnecke:** Writing – original draft, Visualization, Validation, Software, Methodology, Formal analysis, Conceptualization. **Nele L. Bößneck:** Writing – review & editing, Validation, Methodology, Conceptualization. **Alexandra Drebka:** Writing – review & editing, Validation, Methodology, Conceptualization. **Annika J. Scholl:** Writing – review & editing, Validation, Methodology, Conceptualization. **Miriam Degen:** Writing – review & editing. **Nina Ebert:** Writing – review & editing. **Margit Heier:** Writing – review & editing. **Thomas Keil:** Writing – review & editing, Supervision, Resources, Project administration, Funding acquisition, Conceptualization. **Michael Leitzmann:** Writing – review & editing. **Rafael Mikolajczyk:** Writing – review & editing, Project administration, Funding acquisition. **Ilais Moreno Velásquez:** Writing – review & editing. **Nadia Obi:** Writing – review & editing, Investigation. **Phil Pendt:** Writing – review & editing. **Tobias Pischon:** Writing – review & editing, Funding acquisition. **Nahlah Sallam:** Writing – review & editing. **Ben Schöttker:** Writing – review & editing, Data curation. **Margarethe Woeckel:** Writing – review & editing. **Beatrice E. Bachmeier:** Writing – original draft, Visualization, Validation, Supervision, Resources, Project administration, Methodology, Funding acquisition, Conceptualization.

## Ethics approval

The German National Cohort (NAKO) is conducted with the approval of the relevant local ethics committees and is in accordance with national law and with the Declaration of Helsinki of 1975 (in the current, revised version). Written informed consent was obtained from all participants.

## Funding

This project was conducted with data (Application No. NAKO-1052) from the German National Cohort (NAKO) (www.nako.de). The NAKO is funded by the Federal Ministry of Research, Technology and Space (BMFTR) [project funding reference numbers: 01ER1301A/B/C, 01ER1511D, 01ER1801A/B/C/D and 01ER2301A/B/C], Federal States of Germany and the Helmholtz Association, the participating universities and the institutes of the Leibniz Association.

The authors affiliated with Goethe University, Institute of Pharmaceutical Biology, are funded by Dr. Willmar Schwabe GmbH & Co. KG. Open Access funding enabled and organized by Projekt DEAL.

## Declaration of competing interest

The authors affiliated with Goethe University, Institute of Pharmaceutical Biology, are funded by Dr. Willmar Schwabe GmbH & Co. KG. The funder had no role in the conceptualization, design, data collection, analysis, decision to publish, or preparation of the manuscript.

## Data Availability

NAKO data can be obtained via an electronic application portal (www.nako.de/transferhub).
