# Supplementary material for: Anticholinergic burden in the German National Cohort (NAKO): Patterns of anticholinergic drug exposure and utilization
Source: Explor Res Clin Soc Pharm. 2026 Oct 23;24:100869. doi: 10.1016/j.rcsop.2026.100869 (PMC13755950; doi:10.1016/j.rcsop.2026.100869)
Supplement: Supplementary file 1 [file mmc1.docx]

Anticholinergic Burden in the German National Cohort (NAKO): Patterns of Anticholinergic Drug Exposure and Utilization

# Appendix

**Fig. S1**: Overview of baseline recruitment and data collection in the NAKO and the subsequent secondary cross-sectional analysis. AB, anticholinergic burden; ATC, Anatomical Therapeutic Chemical; GABS, German Anticholinergic Burden Scale; NAKO, German National Cohort; OTC, over-the-counter; PZN, Pharmaceutical Central Number.

**Table S1**: Sensitivity analysis of adjusted odds ratios for any and high AB by sex and age group, excluding polypharmacy from the regression model.

|  | **Any burden (GABS score ≥ 1)** | **High burden (GABS score ≥ 3)** |
| --- | --- | --- |
|  | **aOR (95 % CI)** | **aOR (95 % CI)** |
| **Sex** |  |  |
| **Male** | Ref. | Ref. |
| **Female** | 1.25 (1.22-1.28) | 1.73 (1.64-1.83) |
| **Age group** |  |  |
| **≤ 29 years** | Ref. | Ref. |
| **30-39 years** | 1.15 (1.07-1.23) | 1.16 (0.97-1.39) |
| **40-49 years** | 1.67 (1.57-1.76) | 1.90 (1.63-2.20) |
| **50-59 years** | 2.55 (2.41-2.69) | 3.46 (3.00-4.00) |
| **60-69 years** | 3.91 (3.71-4.13) | 4.56 (3.95-5.26) |
| **≥ 70 years** | 4.78 (4.39-5.19) | 4.83 (3.97-5.89) |

AB, anticholinergic burden; aOR, adjusted odds ratio (adjusted for sex, age group, and research site); CI, confidence interval; GABS, German Anticholinergic Burden Scale; Ref., reference.

**Table S2**: Sensitivity analysis of adjusted odds ratios for any and high AB by sex, age group, and number of non-anticholinergic drugs.

|  | **Any burden (GABS score ≥ 1)** | **High burden (GABS score ≥ 3)** |
| --- | --- | --- |
|  | **aOR (95 % CI)** | **aOR (95 % CI)** |
| **Sex** |  |  |
| **Male** | Ref. | Ref. |
| **Female** | 1.05 (1.03-1.08) | 1.48 (1.40-1.57) |
| **Age group** |  |  |
| **≤ 29 years** | Ref. | Ref. |
| **30-39 years** | 1.09 (1.02-1.17) | 1.09 (0.91-1.31) |
| **40-49 years** | 1.44 (1.36-1.52) | 1.58 (1.36-1.84) |
| **50-59 years** | 1.92 (1.81-2.03) | 2.45 (2.12-2.83) |
| **60-69 years** | 2.32 (2.19-2.45) | 2.45 (2.12-2.84) |
| **≥ 70 years** | 2.35 (2.15-2.56) | 2.09 (1.70-2.56) |
| **Number of non-anticholinergic drugs** | 1.30 (1.30-1.31) | 1.28 (1.27-1.29) |

AB, anticholinergic burden; aOR, adjusted odds ratio (adjusted for sex, age group, research site, and number of non-anticholinergic drugs); CI, confidence interval; GABS, German Anticholinergic Burden Scale; Ref., reference.

**Table S3**: Apparent model discrimination (c-statistic) of the main and sensitivity models for any and high AB.

| **Outcome** | **Model** | **C-statistic (95 % CI)** |
| --- | --- | --- |
| Any AB | Sex, age group, research site, polypharmacy | 0.73 (0.73-0.74) |
| Any AB | Sex, age group, research site | 0.64 (0.64-0.64) |
| Any AB | Sex, age group, research site, number of non-anticholinergic medications | 0.73 (0.73-0.73) |
| High AB | Sex, age group, research site, polypharmacy | 0.80 (0.80-0.81) |
| High AB | Sex, age group, research site | 0.66 (0.66-0.67) |
| High AB | Sex, age group, research site, number of non-anticholinergic medications | 0.77 (0.76-0.77) |

AB, anticholinergic burden; CI, confidence interval.

**Fig. S2**: Most frequently used ADs among individuals with high AB (GABS score ≥ 3). Shown are the top 20 ADs, expressed as a proportion of all ADs recorded in this group. Bar colors indicate anticholinergic potency as per the GABS (score 1 (yellow), 2 (orange), or 3 (red)). AB, anticholinergic burden; ADs, anticholinergic drugs; GABS, German Anticholinergic Burden Scale.

**Table S4**: Top 10 most frequently used ADs within each GABS drug score category (scores 1, 2, and 3).

| **GABS score = 1** | | **GABS score = 2** | | **GABS score = 3** | |
| --- | --- | --- | --- | --- | --- |
| **Drug** | **Frequency, *n* (%)** | **Drug** | **Frequency, *n* (%)** | **Drug** | **Frequency, *n* (%)** |
| Metoprolol | 7,899 (4.0) | Opipramol | 1,028 (0.5) | Amitriptyline | 1,105 (0.6) |
| Metformin | 5,069 (2.5) | Quetiapine | 678 (0.3) | Trimipramine | 599 (0.3) |
| Cetirizine | 3,391 (1.7) | Tramadol | 586 (0.3) | Doxepin | 428 (0.2) |
| Citalopram | 1,992 (1.0) | Loperamide | 550 (0.3) | Trospium | 365 (0.2) |
| Venlafaxine | 1,518 (0.8) | Paroxetine | 357 (0.2) | Dimenhydrinate | 296 (0.1) |
| Loratadine | 1,375 (0.7) | Ranitidine | 307 (0.2) | Solifenacin | 281 (0.1) |
| Mirtazapine | 1,214 (0.6) | Carbamazepine | 300 (0.2) | Propiverine | 121 (0.1) |
| Escitalopram | 1,080 (0.5) | Olanzapine | 163 (0.1) | Clomipramine | 81 (< 0.1) |
| Etoricoxib | 960 (0.5) | Theophylline | 130 (0.1) | Tizanidine | 80 (< 0.1) |
| Sertraline | 782 (0.4) | Oxcarbazepine | 65 (< 0.1) | Oxybutynin | 72 (< 0.1) |

Values are presented as *n* (%) of participants in the total analytic sample. ADs, anticholinergic drugs; GABS, German Anticholinergic Burden Scale.

**Fig. S3:** Contribution of OTC medication use to AB ascertainment in the sample. Donut chart showing participants by source of AD exposure: no AD use, prescription (Rx) AD use only, combined Rx and OTC AD use, OTC AD use only, and AD use with unknown prescription status (< 0.1 % of individuals in the total analytic sample; 0.2 % in the AB > 0 group). The enlarged segment displays the corresponding distribution among participants with any anticholinergic burden (AB > 0). The bracket indicates the portion of AB classification sensitive to omission of OTC medication use, comprising participants with OTC-only AD use, whose AB would not be ascertained, and participants with combined Rx and OTC AD use, whose cumulative AB would be underestimated under Rx-only ascertainment. AB, anticholinergic burden; ADs, anticholinergic drugs; OTC, over-the-counter; Rx, prescription.

**Fig. S4:** Proportion of ADs obtained without a prescription (i.e., OTC), overall and by anticholinergic potency category (GABS scores 1-3). Information on the mode of acquisition was unavailable for < 0.2 % of ADs; these were excluded from this analysis. ADs, anticholinergic drugs; GABS, German Anticholinergic Burden Scale; OTC, over-the-counter.
